# Impact of Synthesized Indoloquinoline Analog to Isolates from *Cryptolepis sanguinolenta* on Tumor Growth Inhibition and Hepatotoxicity in Ehrlich Solid Tumor-Bearing Female Mice

**DOI:** 10.3390/cells12071024

**Published:** 2023-03-27

**Authors:** Amany E. Nofal, Elshaymaa I. Elmongy, Engy Abo Hassan, Ehab Tousson, Abdullah A. S. Ahmed, Ibrahim El Tantawy El Sayed, Reem Binsuwaidan, Manar Sakr

**Affiliations:** 1 Zoology Department, Faculty of Science, Menoufia University, Shebin El-Kom 32511, Egypt; amany.nofal@science.menofia.edu.eg; 2 Department of Pharmaceutical Sciences, College of Pharmacy, Princess Nourah bint Abdulrahman University, P.O. Box 84428, Riyadh 11671, Saudi Arabia; rabinsuwaidan@pnu.edu.sa; 3 Chemistry Department, Faculty of Science, Menoufia University, Shebin El-Kom 32511, Egypt; engyebrahim40@gmail.com (E.A.H.); chemist_abdullah_2009@yahoo.com (A.A.S.A.); ibrahimtantawy@science.menofia.edu.eg (I.E.T.E.S.); manarsakr93@gmail.com (M.S.); 4 Department of Zoology, Faculty of Science, Tanta University, Tanta 31511, Egypt; ehabtousson@science.tanta.edu.eg

**Keywords:** CD68, etoposide, APAN, survivin, topoisomerase II, TNF-α

## Abstract

The study evaluated the antitumor efficacy of APAN, “synthesized indoloquinoline analog derived from the parent neocryptolepine isolated from the roots of *Cryptolepis sanguinolenta*”, versus the chemotherapeutic drug etoposide (ETO) in Ehrlich solid tumor (EST)-bearing female mice as well as its protective effect against etoposide-triggered hepatic disorders. APAN showed an ameliorative activity against Ehrlich solid tumor and hepatic toxicity, and the greatest improvement was found in the combined treatment of APAN with ETO. The results indicated that EST altered the levels of tumor markers (AFP, CEA, and anti-dsDNA) and liver biomarker function (ALT, AST, ALP, ALB, and T. protein). Furthermore, EST elevated CD68 and anti-survivin proteins immuno-expressions in the solid tumor and liver tissue. Molecular docking studies were demonstrated to investigate their affinity for both TNF-α and topoisomerase II as target proteins, as etoposide is based on the inhibition of topoisomerase II, and TNF-α is quite highly expressed in the solid tumor and liver tissues of EST-bearing animals, which prompted the authors’ interest to explore APAN affinity to its binding site. Treatment of mice bearing EST with APAN and ETO nearly regularized serum levels of the altered parameters and ameliorated the impact of EST on the tissue structure of the liver better than that by treatment with each of them separately.

## 1. Introduction

The global incidence of cancer is consistently increasing, endangering the lives of immeasurable individuals. Cancer is instigated by a complex blend of environmental, food, water, air pollution, and genetic factors, which contributes to its prevalence [1]. The resistance of cancer cells to chemotherapy is one of the severe causes of cancer-related demise. Hence, innovative strategies to improve and handle the circumvention of drug clinical outcome reactions are needed. Thus, a mixture of chemotherapeutic protocols is the standard [2,3]. The primary definition of the Ehrlich tumor is that it is an unexpected murine mammary adenocarcinoma that could be employed as a transplantable tumor prototype for the simple investigation of the antineoplastic effects of several chemical substances [4]. Though, it induced nephrotoxicity, hepatotoxicity, and oxidative stress [5,6]. Etoposide (ETO) is a chemotherapeutic drug that is used against various cancers such as glioblastoma multiforme, lymphoma, leukemia, and testicular cancer [7]. Etoposide is a semi-synthetic subordinate of podophyllotoxin [4′demethylepipodophyllotoxin-9-(4, 6-*O*-ethylidene)–β–d-glucopyranoside, a non-alkaloid lignan secluded from the dried-out roots, and rhizomes of Podophyllum peltatum (Berberidaceae) [8]. ETO is based on the inhibition of topoisomerase II (TOP2) action, which is used for human malignancy treatment, by preventing the TOP2–DNA cleavable compound, leading to DNA damage and then apoptosis [9]. TOP2 is an enzyme that has vital functions including transcription, DNA replication, and chromosome segregation [10]. Malignant cells depend on TOP2 more than normal healthy cells, as they divide more swiftly [11].

**Figure 1:**
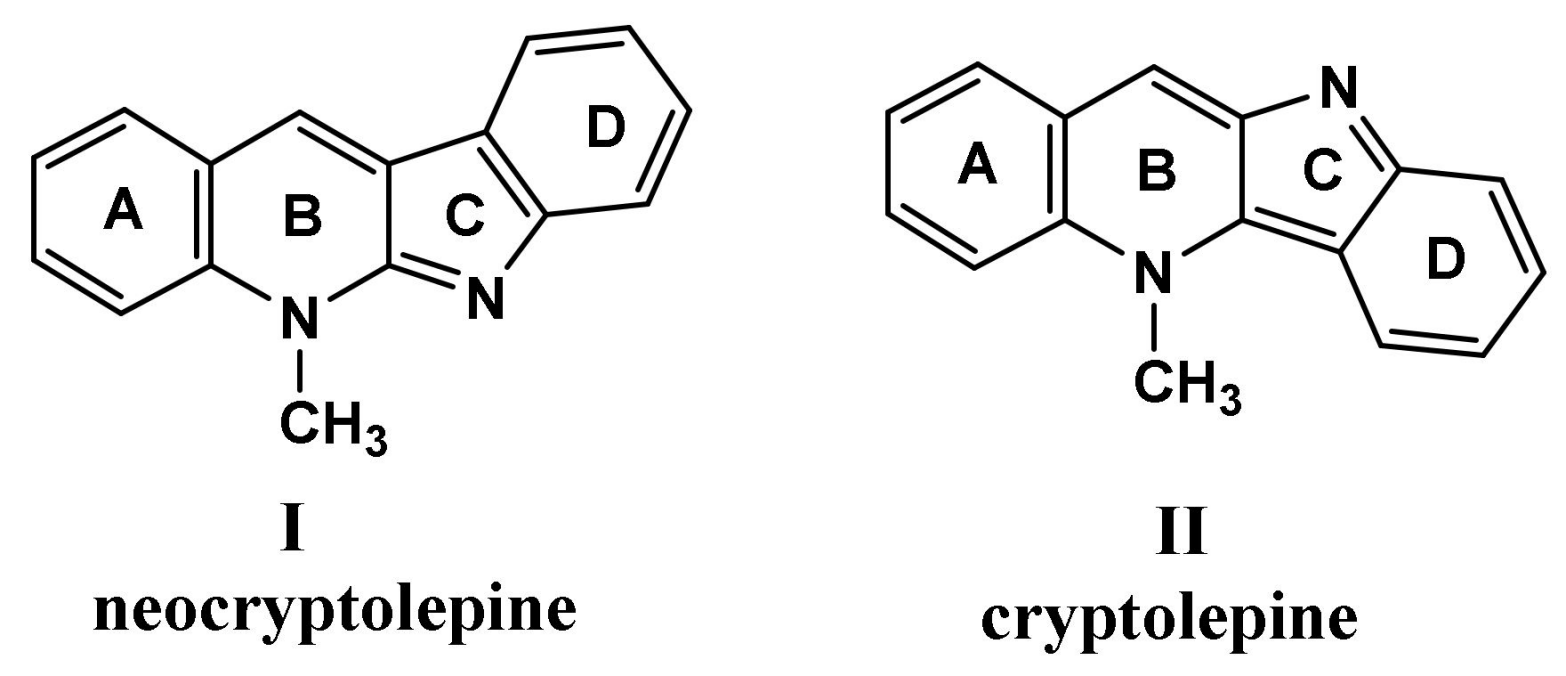
Indoloquinolines from Cryptolepis sanguinolenta.

It is worthy to note that nature is an enriched source of valuable pharmaceutical cores as heterocyclic compounds which are used for curing many diseases [12,13,14]. Indoloquinolines isolated from *Cryptolepis sanguinelenta* gained great interest in medicinal chemistry; among them, the alkaloid neocryptolepine I and its regio-isomer cryptolepine II (Figure 1) received much attention by researchers [15]. These two alkaloids have tunable biological applications in medicinal chemistry as anticancer, antimicrobial, antimalarial and anti-inflammatory as well as affinities towards DNA interaction and DNA topoisomerase inhibition [16,17]. Owing to the biological importance of these two alkaloids, several structural modifications were established to improve their potency and selectivity to cancer cells [18,19].

**Figure 2:**
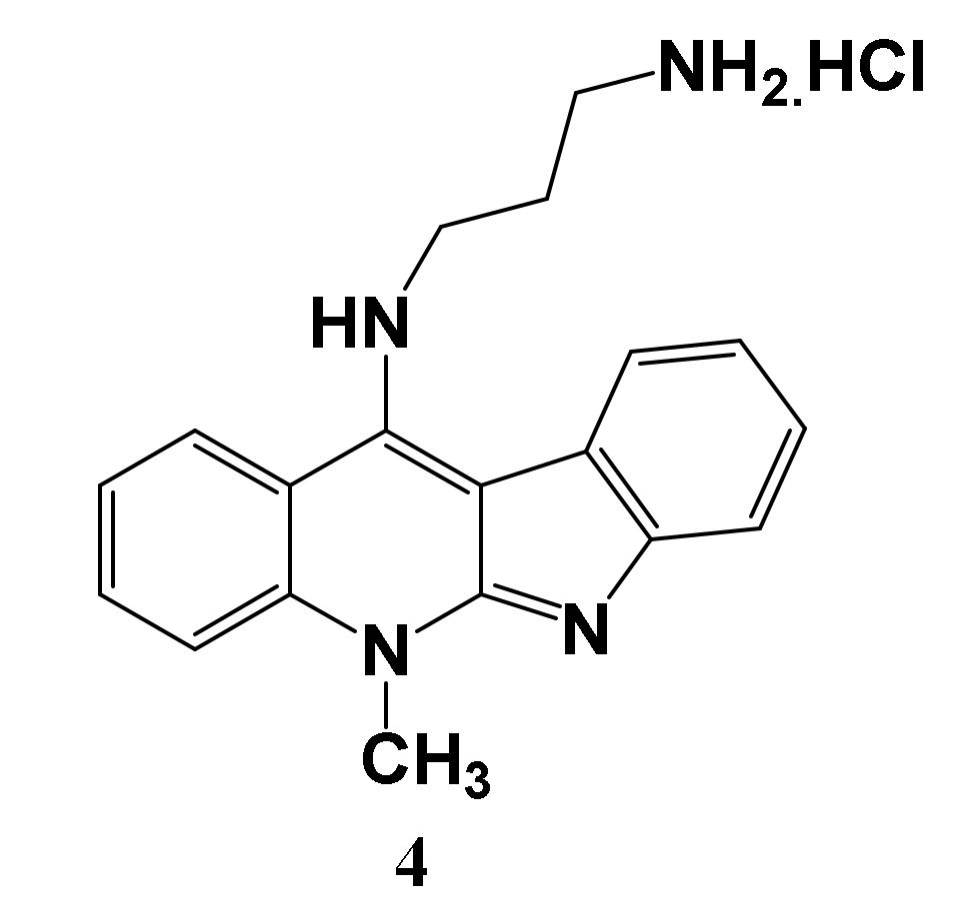
Structure of 11-Aminopropylamino neocryptolepine hydrochloride salt (APAN).

The in vitro anticancer activity of the free 11-aminopropylaminoneocryptolepine (APAN) **3** (Figure 2) was recently reported [19], and it exhibited significant anticancer activity against a breast cancer cell line (MDA-MB-453) with an IC_50_ of 0.50 µM. These results encouraged the authors to further explore the antitumor activity in vivo of **3** on the animal model. In this study, we have administrated compound **3** as a hydrochloride salt to enhance the aqueous solubility as well as the bioavailability.

## 2. Materials and Methods

### 2.1. Chemistry

Nuclear magnetic resonance spectroscopy (NMR) analysis was executed with Bruker Bruker magnet system 400,54 Ascend/R (USA) of 400 MHz and 101 MHz for ^1^H NMR and ^13^C NMR, respectively, as well as FT-IR spectroscopy was performed with Alpha-Bruker ATR mode (USA) at the Faculty of Science, Zagzig University (Zagzig, Egypt). The commercial starting material 1.3-diamino propane was purchased from Sigma Aldrich and the synthesized mother core of 11-chloroneocryptolepine **1** and its free amine **2** were attained according to methods in the literature [17]. 

General procedure for the synthesis of ammonium chloride of aminopropylamino neocryptolepine, APAN (**4**), was followed. Under the stirring condition, an excess amount of 1.0 M HCl was added to a solution of the free amine of neocryptolepine **3** dissolved in 4 mL methanol till the formation of yellowish green precipitate **4**, followed by filtration and drying. N1-(5-methyl-5H-indolo [2,3-b]quinolin-11-yl)propane-1,3-diaminehydrochloride (4), a yellowish green solid with yield (0.63 g, 70%), and results of FT-IR (KBr) cm^−1^ are: 3425(NH), 3211 (NH_2_), 3051 (CH_Ar_), 2964 (CH), 1595(C=C_Ar_), and 1219(C-C). ^1^H NMR (CDCl_3_, 400 MHz, δ ppm) results are: 1.90(m, 2H, CH_2_); 2.48 (t, 2H, CH_2_, *J* = 8 Hz); 3.97 (br.m, 2H, CH_2_, *J* = 8 Hz); 4.26 (s, 3H, N-CH_3_); and 7.36–7.93 (m, 8H, CH_Ar_). ^13^C NMR (CDCl_3_, 101 MHz, δ ppm) results are: 33.34, 37.58, 52.45, 55.01, 102.25, 110.81, 115.20, 115.53, 120.09, 121.12, 121.63, 122.32, 122.74, 124.05, 124.19, 124.67, 124.90, 125.74, 131.15, 134.74, 137.06, 139.18, and 146.89. 

### 2.2. Modeling Study

Molecular docking studies were performed using MOE [Chemical Computing Group Inc. Molecular Operating Environment (MOE); Chemical Computing Group Inc.: Montreal, QC, Canada, 2014; 09.].The target protein was downloaded from the protein data bank code 2AZ5 for TNF-α and 3FOE for topoisomerase II. Ligand and protein structural optimizations were applied by calculating partial charges, 3D protonation, and strands correction followed by energy minimization [20]. Ligand was selected as the placement guide. Pharmacophore annotations were excluded. Browsed database of the investigated compound was in mdb format. The gradient for energy minimization was 0.05, and MMFF94X was the force field by default [21]. 

### 2.3. In Vivo Biological Assays

Etoposide (ETO) was fabricated by Ebewe Pharma-Egypt and was sold as a colorless solution and stockpiled in the ward or the pharmacy; the injection was reserved in a cool dry place where the temperature stayed below 25 °C and secured from light according to the leaflet. ETO was given as intraperitoneal infusions at 50 mg/kg body weight/twice a week [22].

Lethal dose (LD_50_) of APAN. The LD_50_ value for APAN was calculated through conventional methods. APAN was dissolved in distilled water at different doses, including 50, 100, 150, 200, 250, 300, 350, 400, 450, and 500 mg/kg bw of mice. LD_50_ was estimated by recording the rate of mortality after 24 h. The approximate LD_50_ of APAN was determined by probit analysis equation in accordance with Finney [23]. The selected dose was 1/10th of LD_50_ = 300 (nearly 30 mg/kg bw).

#### 2.3.1. Animals

Eighty female Swiss albino adult mice weighing 25–28 g were acquired from the Egyptian Organization for Biological Products and Vaccines breeding unit (Cairo, Egypt). The mice’s diet was tap water ad libitum and supplied commercial standard pellet diet. During the experimental period, rearing and treatment of mice were conducted according to the Faculty of Science, Tanta University rules for animals, which was agreed upon by the Institutional Animal Care and Use Committee (IACUC-SCI-TU-0047).

#### 2.3.2. Ehrlich Solid Tumor (EST) Induction

Mice bearing Ehrlich ascites carcinoma (EAC) were obtained from the Egyptian National Cancer Institute, Cairo University, Egypt and used as the basis for EAC cells to induce solid tumors in the investigational mice; a quantity of 0.2 mL of ascitic fluid was aspirated from EAC mice and diluted with physiological saline and EAC cells were counted. Around 2.5 to 3 million EAC cells were subcutaneously inoculated in the left thigh of the lower limb of each animal [24].

#### 2.3.3. Experimental Design

After one week of acclimation, overall 80 female mice were ambiguously and equally allotted into eight groups, each consisting of 10 mice. Mice that were not injected with anything were kept as the control group (Con). The animals that were intramuscularly (im) injected inside the left hip with APAN (30 mg/kg bw/twice a week) for two weeks were categorized in one group (APAN). The mice that were injected intraperitoneally (ip) with ETO for two weeks (50 mg/kg bw/twice a week) [22] were categorized as one group (ETO). The mice that were injected with both the APAN analog (15 mg/kg bw/im, twice a week) inside the hip and ETO (25 mg/kg bw/ip, twice a week) for two weeks were categorized as one group (APAN+ETO). The mice that were subcutaneously (sc) injected inside the left hip with 2.5 million cells of EAC for each mouse diluted in physiological saline to initiate tumor for 2 weeks [24] were categorized in one group (EST). The animals that were sc injected with 2.5 million cells of EAC for each mouse diluted in saline to initiate the tumor for 2 weeks till the development of EST and then treated with the APAN analog im inside the tumor for another two weeks were categorized as one group (mono treatment, EST+APAN). The mice that have been injected sc with 2.5 million cells of EAC per mouse diluted in saline to initiate the tumor and left for 2 weeks till the growth of the solid tumor and then treated with ETO for another 2 weeks were categorized as one group (mono treatment, EST+ETO). The mice that have been injected sc with 2.5 million cells of EAC to induce EST and left for 2 weeks and then treated with both APAN and ETO with half doses of each were categorized into one group (dual treatment, EST+APAN+ETO).

#### 2.3.4. Tumor Weight and Volume

After tumor inoculation, the left thigh dimensions of the lower limb of different groups were weighed on the digital electronic balance and using a two-end electronic digital caliber for its measurement (Switzerland), each other day till the 30th day, the end of the experiment. Tumor size was then calculated as reported by Goto et al. [25] using the formula:

Volume (mm^3^) = Length × (width)^2^/2

#### 2.3.5. Samples

After the period of the experiment finished, the mice were euthanized by cervical dislocation and subjected to necropsy. Samples of blood were collected individually from the inferior vena cava of each mouse and allowed to form clots, after that were centrifuged at 4000 rpm for 15 min, and then sera were kept at −80 °C until use [26]. The obtained tumors of the left thigh muscles and liver were prepared for subsequent analysis.

#### 2.3.6. Histopathological Examination

Small pieces of the left thigh muscles with or without tumor masses and liver were carefully removed from all groups of the experiment and rinsed in an isotonic solution and then fixed in 10% neutral formalin. Tissue paraffin blocks were prepared by the routine method, and then 5 μm sections were stained with hematoxylin and eosin [27].

#### 2.3.7. Immunohistochemical Determination of Tumor Cell Proliferation and Apoptosis

Ehrlich tumor and liver sections were routinely processed by using a standard avidin-biotin-peroxidase complex system in accordance with the manufacturer’s instructions for cell macrophage (CD68) according to Tousson et al. [28], tumor necrosis factor-alpha (TNF-α) according to Calabrese et al. [29], and survivin according to Das et al. [30]. In order to quantify the histopathological and IHC results, 1 × 10^3^ cells from at least eight different sections were investigated using Image J software (ImageJ bundled with 64-bit Java 8, Version 1.53t, NIH, Bethesda, MD, USA) [31].

#### 2.3.8. Biochemical Assays

Serum alpha-fetoprotein (AFP) was detected by using an automated quantitative enzyme-linked fluorescent assay (ELFA) with mini-VIDAS^®^AFP (Biomerieux, Marcy-L’Etoile, France) in accordance with Jang et al. [32]. The alteration of the color representing the CEA concentration was decided by using microplate ELISA reader Biotek elx808 at the wavelength 450 nm [33]. Enzyme immunoassay kits (EIA) were used for recognition of anti-dsDNA antibodies, obtained from Helix Diagnostics (West Sacramento, CA, USA), according to the kit supplier’s protocol (Demeditec Diagnostics, Kiel, Germany) [34]. The technique described by Saggu et al. [35] was modified to analyze the activities of aspartate aminotransferase (AST) and alanine aminotransferase (ALT) by using the commercial kit bought from Bio diagnostic, Egypt. Alkaline phosphatase (ALP) activity was measured by using a commercial kit (BioMérieux Co., Lyon, France), conferring to [36]. The method described by Doumas [37] was used to measure albumin levels by using a commercial kit (Diamond, Alexandria, Egypt). Additionally, the total protein level was measured following Lowry et al. [38], using a commercial kit (Diamond, Egypt).

### 2.4. Statistical Analysis

The data of different groups were expressed as the mean ± SE. The differences between groups were evaluated using a statistical package of social science (SPSS) software, version 22 for Windows. One-way analysis of variance (ANOVA) and the least noteworthy difference (LSD) for post hoc analysis were used for several comparisons. Statistical significance was considered when the value was *p* < 0.01.

## 3. Results

### 3.1. Chemistry

**Scheme 1:**
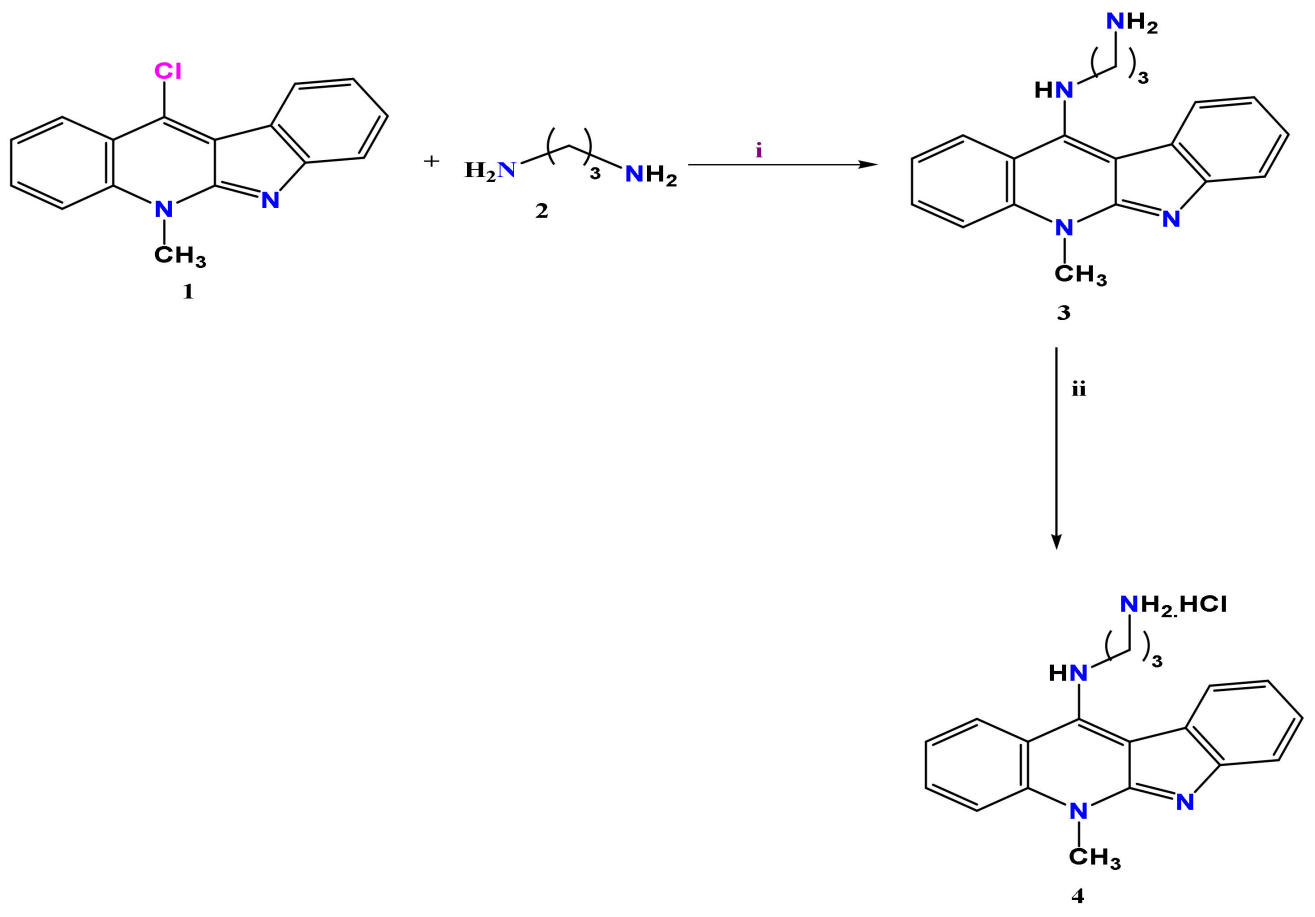
Synthesis of 11-aminopropylamino neocryptolepine and its hydrochloride salt (APAN). Reagents and conditions: (i) 3 times excess, DMF, reflux, 2 h. (ii) excess amount of 1M of HCl, methanol.

The challenge to find a potent, selective, and safe new anticancer drug is one of the main objectives for many researchers. Many reports discussed the biological importance of natural product alkaloids as anticancer agents [12]. In this context, neocryptolepine proved to be a promising natural product lead in drug discovery and development. Many neocryptolepine analogs bearing a pharmacophoric amine moiety on the different core positions were recently synthesized and screened for their anticancer activity in vitro including analog **3**. On the other hand, little is known about the in vivo study on amine-bearing neocryptolepine scaffolds. In this study, we have prepared the target compound **4**, following our previously reported method, starting from the key intermediate 11-chloroneocryptolepine **1** with an excess amount of 1,3-diamino propane to afford the free amine structure **3** via nucleophile aromatic substitution reaction (SN_Ar_). The free amine was further dissolved in methanol followed by the addition of 1M of HCl dropwise till the formation of the corresponding salt **4**, as given in Scheme 1. The salt structure was elucidated using spectroscopic tools (c.f., experimental data for more details).

### 3.2. Modeling Studies

Molecular docking studies were performed aiming to support the biological results towards the target proteins TNF α and topoisomerase II. Selection of the targets was based on the mode of action of the reference ‘etoposide,’ which acts as a topoisomerase II inhibitor, and TNF-α is an especially pleiotropic cytokine that has a crucial job in protection, homeostasis, and immunity and is quite highly expressed in a solid tumor and liver tissues of EST animals, which prompted the authors’ interest to explore APAN affinity to its binding site. 

**Figure 3:**
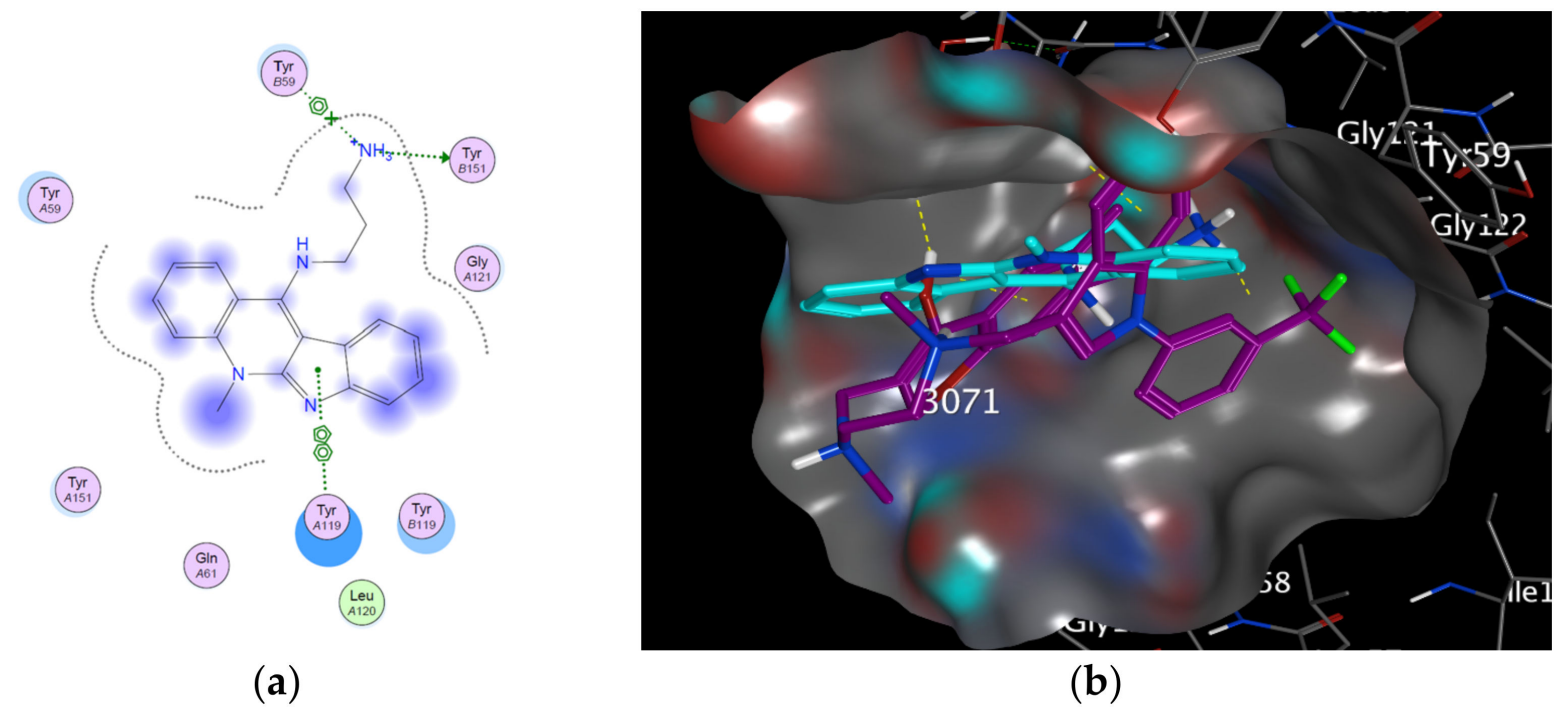
Docking of APAN at the target protein pdb:2AZ5; (**a**) 2D interaction and chemical bonding; (**b**) surface view for APAN complexed with the co-crystalized ligand at the site of interaction.

Regarding TNF-α, sixteen contact amino acid residues were demonstrated in the active site at the interface of dimer, including Leu57, Tyr59, Gln61, Tyr119, Gly122, Leu120, Ser60, Gly121, and Tyr151 from chain **A** and Leu57, Tyr59, Ser60, Tyr119, Leu120, Gly121 and Tyr151 from chain **B**. Six tyrosine amino acid residues were involved, among which Tyr119 is the most crucial residue and its chi-1 angles rotate to form a dimer (chains **A** and **B**) by accommodating compound binding. Detailed molecular interaction pattern demonstrated hydrogen bonding and *pi-*cation and *pi* hydrophobic interactions; the latter type of interactions was between APAN and the receptor pocket key aminoacid Tyr119, recording binding affinity (−5.0704) and RMSD (2.08865). It is worth mentioning that APAN was complexed with the co-crystalized ligand at the pocket of interaction, sharing the same interaction with the key amino acid TYR119 (Figure 3).

**Figure 4:**
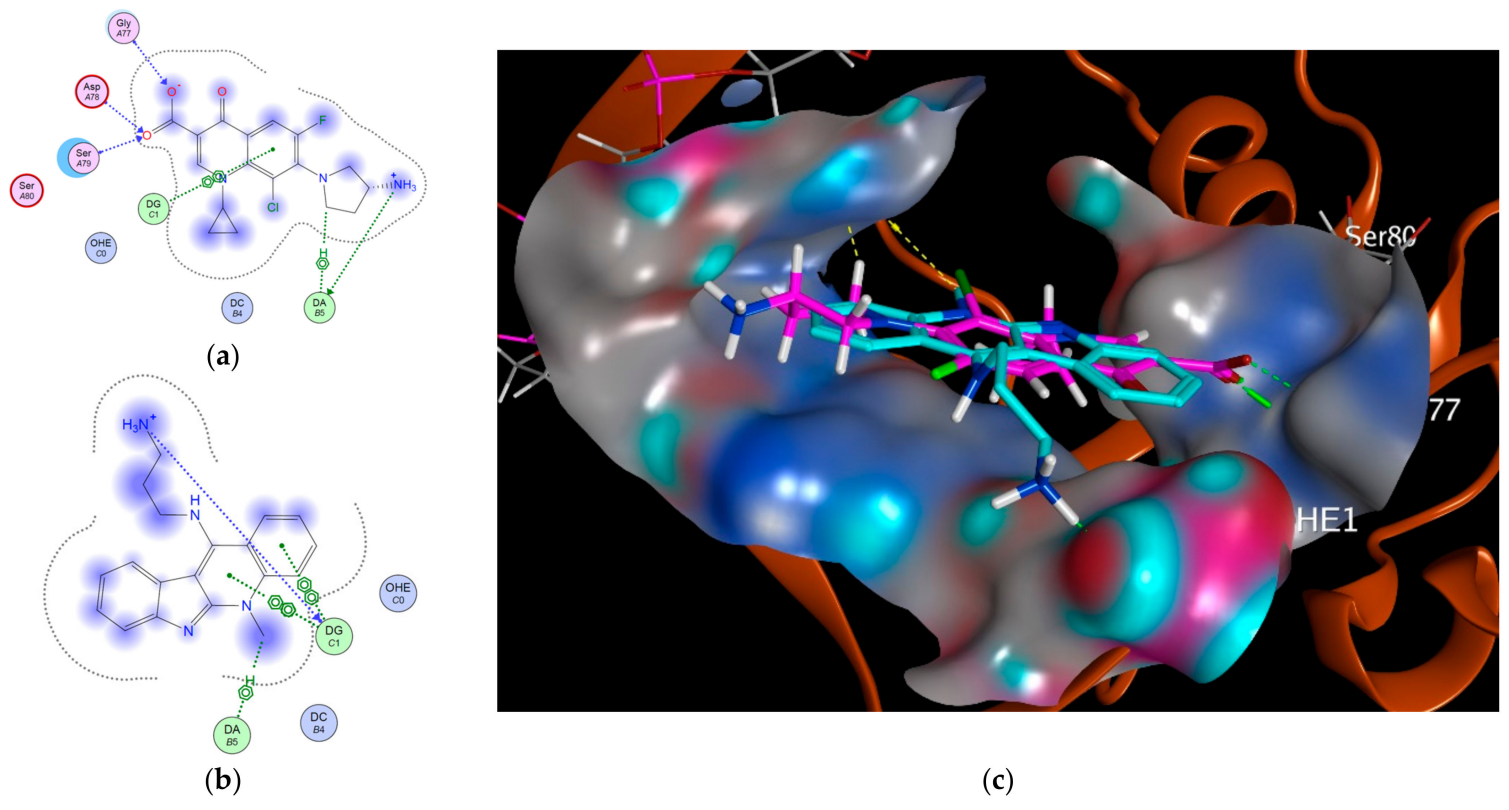
Docking at the target protein pdb:3FOE; (**a**) 2D interaction of protein co-crystalized ligand NFX; (**b**) 2D interaction of APAN; (**c**) surface view of APAN in cyan sticks complexed with the co-crystalized ligand NFX in pink sticks at the site of interaction (H-bonds in green color and hydrophobic pi interactions in yellow color).

Regarding topoisomerase II, the proposed binding mode of APAN showed an affinity value of −5.5996. It showed one hydrogen bond interaction in addition to *pi* interactions, and the planer aromatic system of substituted neocryptolepine showed aromatic stacking interactions, while the terminal aminopropylamino moiety was directed at the DNA-minor groove (Figure 4). Docking results including residues involved in the interactions as well as the types of bonding are tabulated in Table 1.

### 3.3. Tumor Weight and Volume

**Figure 5:**
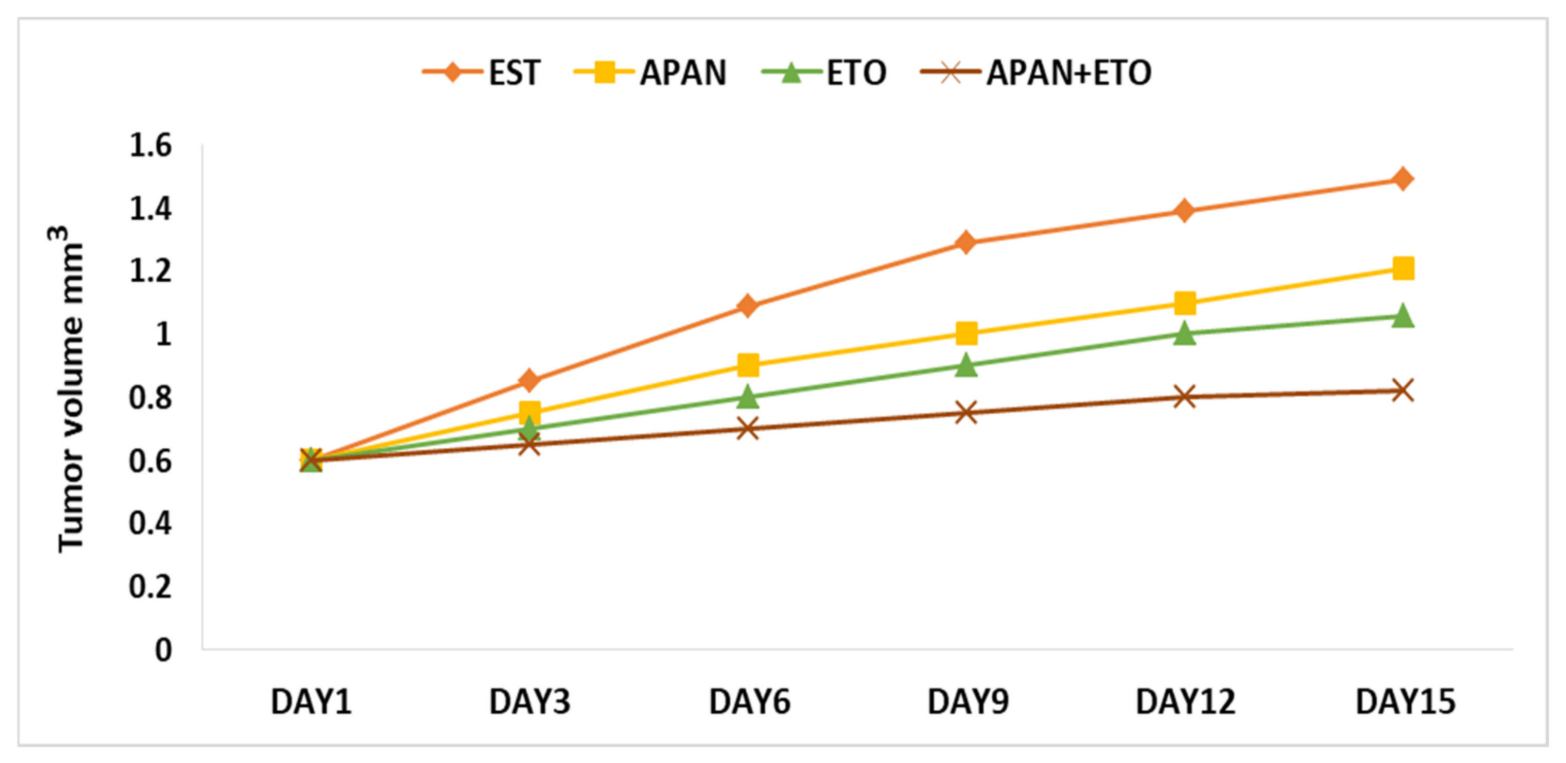
Chart representing changes in tumor volume vs. days over the course of mono (APAN or ETO) and dual treatment (APAN+ETO) against Ehrlich solid tumor-bearing mice (EST).

The effect of the treatment of solid tumor implanted in mice with APAN or/and ETO on the progress and proliferation of subcutaneously injected EST was evaluated by their growth-dependent variation at tumor weight and volume in different groups after 14 days of EST injection. Table 2 demonstrated a significant reduction (*p* < 0.01) in tumor weight and volume in different treatment groups (EST+APAN, EST+ETO, and EST+APAN+ETO) compared with the EST-non-treated group at the end of the experiment. The maximum decrease ratio was seen in the dual treatment group compared with EST and mono treatment groups. Figure 5 showed a graph representing changes in tumor size vs. days over the course of mono and dual treatment.

### 3.4. Histopathological Observations

**Figure 6:**
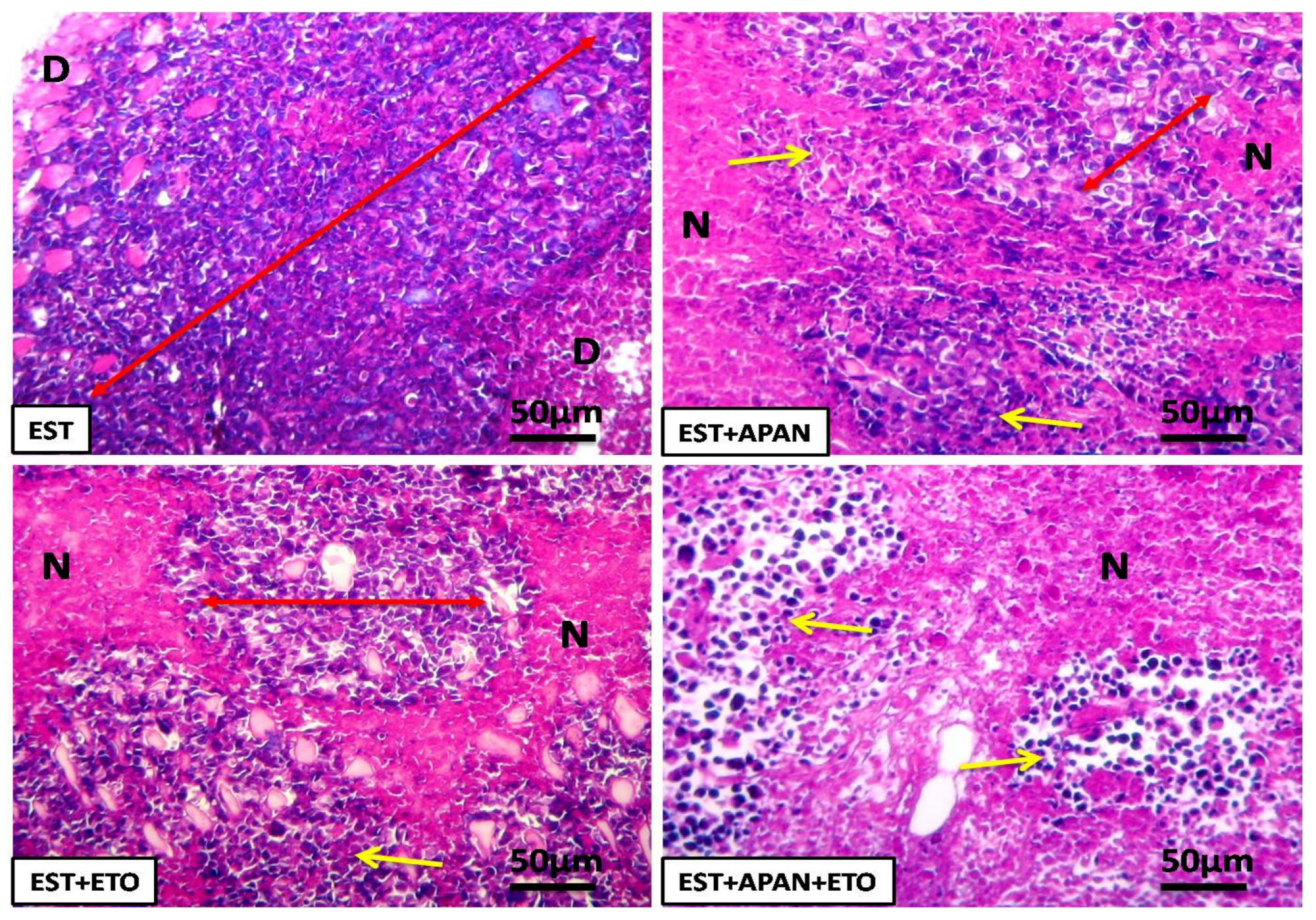
Light microscopic photomicrographs of H and E solid tumor sections of female mice in the different experimental groups. EST group shows hyperchromatic tumor cell populations (double-headed arrow) and irregular degenerated muscle fiber (D). EST mice treated with APAN or ETO reveal a moderate degree of improvement with the remaining tumor cell population (double-headed arrow), mild inflammatory cell infiltrations (thin arrows), and diffuse necrosis of skeletal muscle tissue (N). EST mice treated with both APAN+ETO reveal strong diffuse necrosis of skeletal muscle tissue (N) around the remaining tumor cells population (double-headed arrow) and marked inflammatory cell infiltrations (thin arrows). Scale bar, 50 µm.

EST-bearing mice showed nearly complete infiltration of the skeletal muscles with irregular eosinophilic tumor cells with marked hyperchromatic nuclei, edema, and irregular homogenized degenerated muscle fiber. EST mice treated with APAN or ETO revealed a moderate degree of improvement with the remaining tumor cell population, mild inflammatory cell infiltrations, and mild diffuse necrosis of skeletal muscle tissue. EST treated with both APAN and ETO revealed strong diffuse necrosis of skeletal muscle tissue around the remaining tumor cells population and marked inflammatory cell infiltrations in skeletal muscle tissue (Figure 6).

**Figure 7:**
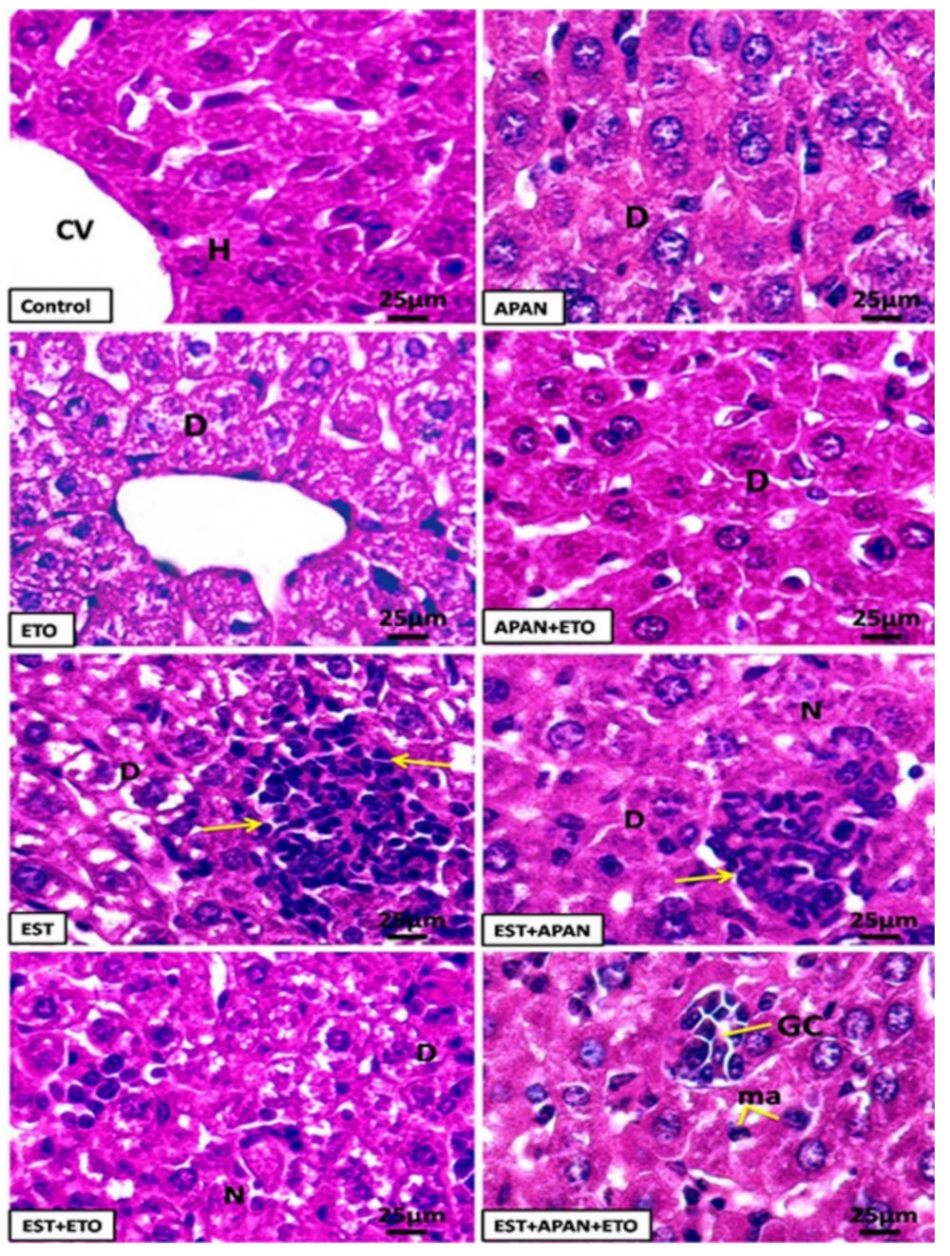
Photomicrographs of liver H and E sections of female mice in the different experimental groups. The control group reveals a normal structure of hepatocytes (H) with normal central veins (CV). APAN, ETO, and APAN+ETO groups display mild cytoplasmic vacuolization and degeneration of hepatocytes (D). EST group shows a strong degeneration with cytoplasmic vacuolization (D) and marked inflammatory cells infiltrations with irregular hyperchromatic tumor cell population (thin arrows). EST mice treated with APAN reveal a modest degree of improvement in hepatocyte damage, inflammatory cell infiltrations with some irregular hyperchromatic tumor cells population (thin arrows), giant cell (GC), diffuse necrosis of hepatic tissue (N), and mild degeneration of hepatocytes (D). EST mice treated with ETO show mild diffuse necrosis of hepatic tissue (N), degeneration of hepatocytes with a marked cytoplasmic vacuolization (D), and giant cell (GC). EST mice treated with both APAN+ETO reveal a significant degree of improvement in hepatocyte damage with giant cell (GC) and enlarged macrophage cells (ma). Scale bar, 25 µm.

Liver sections of the control group exposed the standard structure of the hepatocytes, which are polygonal cells with eosinophilic cytoplasm, prominent round nuclei, and a few spaced blood sinusoids with Kupffer cells in between the hepatic cords. Mice that received APAN or APAN+ETO revealed a faint histological change in the structure of the liver, mild intracytoplasmic vacuolization, and degeneration of hepatocytes. The ETO group revealed a mild change in the histological structure of the hepatocytes with a mild change in the arrangement of Kupffer cells. Additionally, liver sections of the EST group showed marked degeneration and inflammatory cells with irregular hyperchromatic tumor cell population in hepatic cords. Liver sections of treated EST mice with the APAN group exposed a reasonable degree of betterment in hepatocytes with some irregular hyperchromatic tumor cell population, cellular infiltrations, multinucleated giant cells, and diffuse necrosis of hepatic tissue. Liver sections of treated EST with ETO group exposed a mild degree of betterment in hepatocytes, where marked microvacuolated hepatocytes, mild diffuse necrosis of hepatic tissue, and giant cells were observed. On the other hand, liver sections of treated EST animals with both APAN and ETO exposed a significant mark of improvement, and the liver structure resembles the control with multinucleated giant cells, and enlarged macrophage cells were observed (Figure 7).

### 3.5. Immunoreactivity of CD68, TNF-α, and Survivin

The histopathological and immunohistochemical scores for the EST mice treated with mono or/and a combination of APAN and ETO are represented in Table 3. The obtained data revealed that cell degeneration, necrosis, cellular inflammatory infiltration, tumor cells, cytoplasmic microvacuolation, giant cell, and immuno-expressions of CD68, TNF-α, and survivin significantly decreased in mono and dual treatment with APAN or/and ETO groups in comparison to the EST mice.

**Figure 8:**
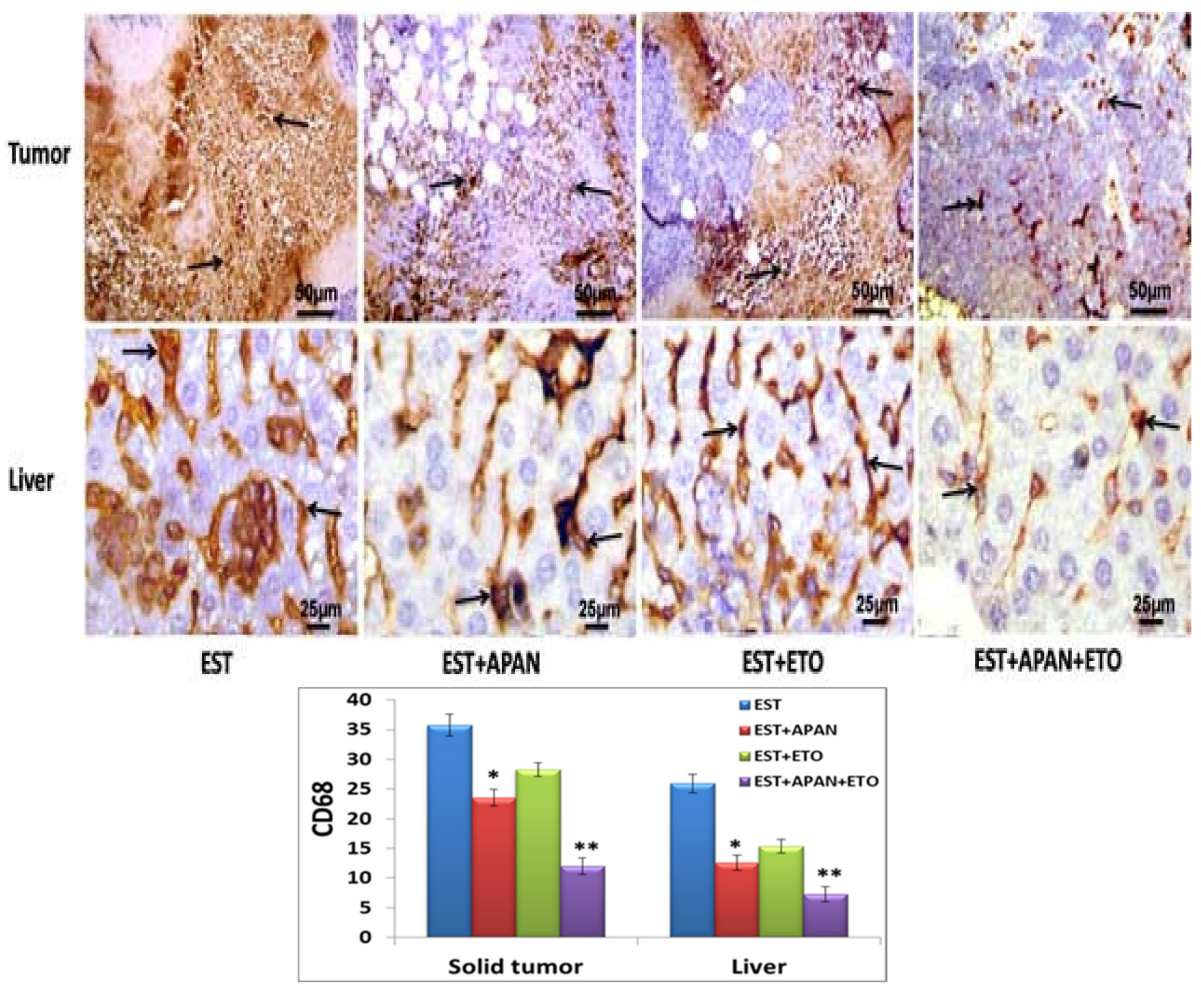
Photomicrographs and chart of CD68 immunohistochemical expression (arrows) of Ehrlich solid tumor-bearing mice (EST) after mono (EST+APAN or EST+ETO) and dual treatment (EST+APAN+ETO). Scale bar of solid tumor = 50 µm and liver = 25 µm, (*) indicates significantly different, (**) indicates the maximum decrease ratio compared to the EST group, and *p* < 0.01.

The positive immunohistochemical reactivates of Cluster of Differentiation 68 (CD68), proinflammatory cytokine tumor necrosis factor-alpha (TNF-α), and antiapoptotic protein survivin (SV) appeared as brown color in the solid tumor from the left thigh of the lower limb and liver tissues in different treatment groups. CD68 is an immunohistochemical marker of macrophage cells, which have various functions in immunity, inflammation, and tumor biology. The CD68-positive immunoexpression macrophages were significantly greater in a solid tumor and liver tissues of the EST group than in EST-treated mice with APAN or ETO groups; moreover, it was very faint in all other groups (Figure 8, Table 3). 

**Figure 9:**
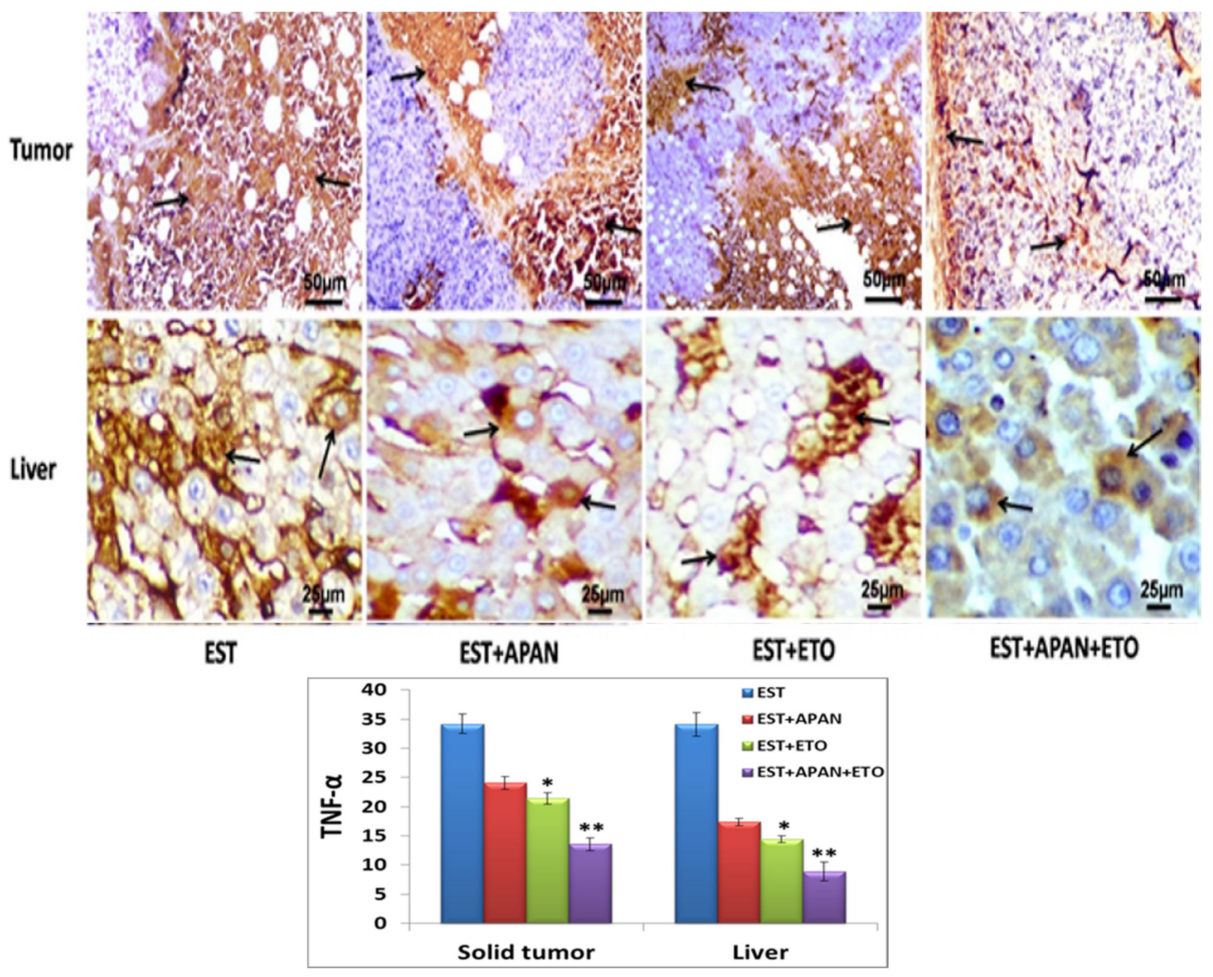
Photomicrographs and chart of TNF-α immunohistochemical expression (arrows) of Ehrlich solid tumor-bearing mice (EST) after mono (EST+APAN or EST+ETO) and dual treatment (EST+APAN+ETO). Scale bar of solid tumor = 50 µm and liver = 25 µm, (*) indicates significantly different, (**) indicates the maximum decrease ratio compared to the EST group, *p* < 0.01.

**Figure 10:**
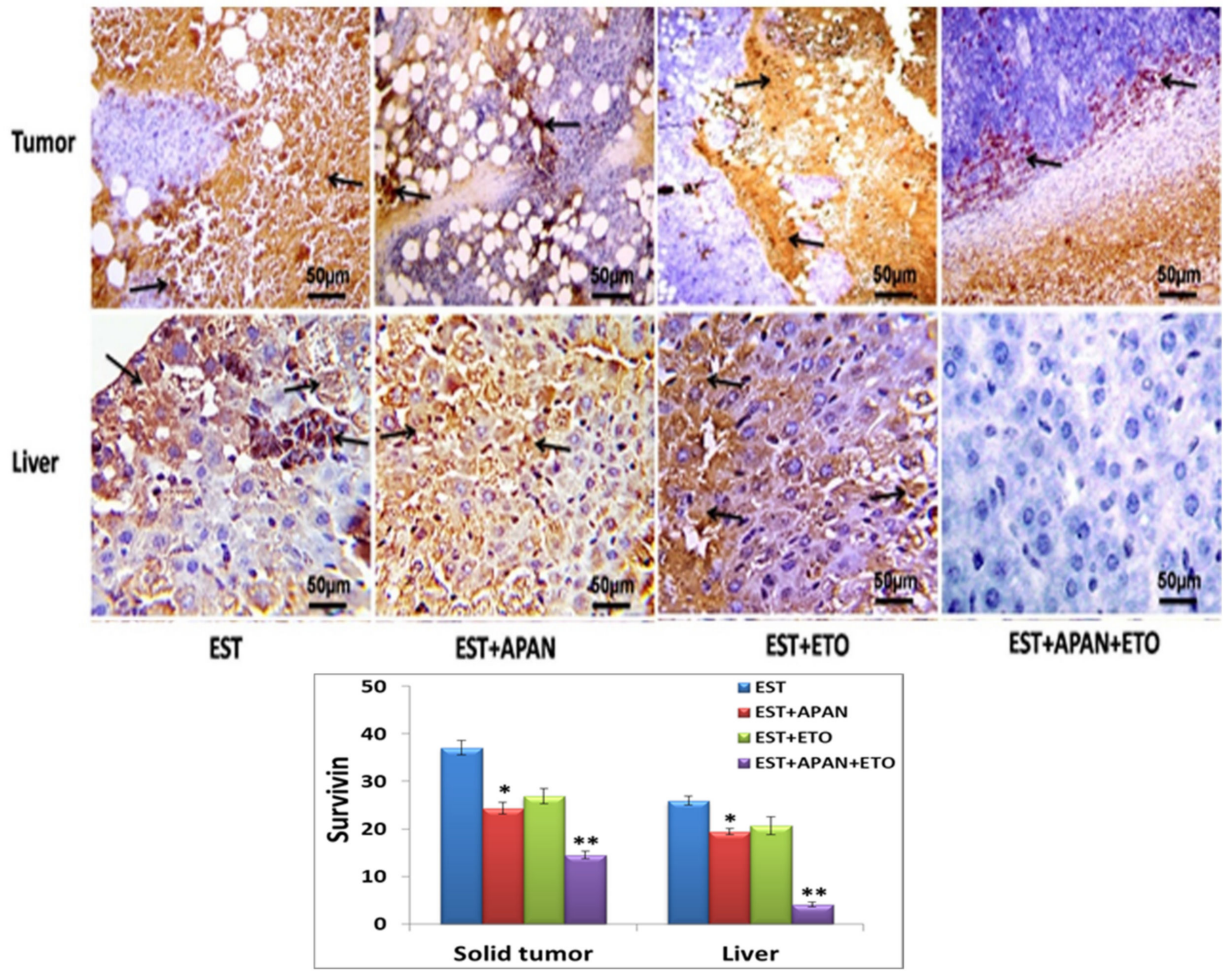
Photomicrographs and chart of survivin immunohistochemical expression (arrows) of Ehrlich solid tumor-bearing mice (EST) after mono (EST+APAN or EST+ETO) and dual treatment (EST+APAN+ETO). Scale bar of solid tumor and liver = 50 µm, (*) indicates significantly different, (**) indicates the maximum decrease ratio compared to the EST group, *p* < 0.01.

TNF-α is an especially pleiotropic cytokine that has a crucial job in protection, homeostasis, and immunity. In the present research, TNF-α was quite highly expressed in the solid tumor and liver tissues of EST animals and was markedly downregulated in EST-treated with mono and/or dual treatment groups in comparison to the EST mice. The maximum reduction was seen in the dual-treatment group compared with EST and mono-treatment groups (Figure 9, Table 3). The EST mice induced a rise in survivin immune reactivity tumor, and the treatment of EST with both APAN +ETO was more effective rather than treatment with one of them alone to reduce these survivin expressions (Figure 10, Table 3).

### 3.6. Serum Tumor Marker

Table 4 shows the changes in serum levels of tumor markers in the different groups under study. It revealed a significant (*p* < 0.01) surge in the levels of alpha-fetoprotein (AFP), carcinoembryonic antigen (CEA), and antibodies to double-stranded DNA (anti-ds DNA) in the EST group compared with control animals. The mice-bearing solid tumors after treatment with APAN, ETO, or both of them had significantly (*p* < 0.01) decreased AFP, CEA, and anti-ds DNA compared with the EST group and revealed that dual treatment is the best option for EST treatment.

### 3.7. Liver Function Test

Table 5 reveals that alanine aminotransferase (ALT), aspartate aminotransferase (AST), and alkaline phosphatase (ALP) in serum were significantly elevated, while albumin (ALB) and total protein were significantly reduced in the EST group in comparison with the control group. The EST+APAN group showed no change in ALT level, a noteworthy decrease in AST and ALP, as well as a slight increase in ALB and total protein compared with the EST group. The EST+ETO group showed a significant decrease in ALT, AST, and ALP, while showing a slight increase in ALB and total protein levels in comparison with the EST group. The EST+APAN+ETO group displayed a significant decrease in ALT, AST, and ALP, while showing a noteworthy increase in ALB and total protein levels in comparison with the EST group. 

## 4. Discussion

Cancer is one of the most fatal diseases, and one of the prime reasons of death worldwide characterized by unscheduled and uncontrolled cellular proliferation [39]. Akin to breast cancer in humans, Ehrlich carcinoma personifies an undifferentiated carcinoma that begins as hyperdiploid, has a high ability for transplantation, does not relapse, proliferates swiftly, has a short-term lifespan, is entirely malignant, and without a tumor-specific transplantation antigen [40]. This study intended to investigate the effect of neocryptolepine analog (APAN) alone or/and combined with Etoposide (ETO) as co-treatment for Ehrlich solid tumor (EST)-caused hepatic injury in female mice and its synergism and ability to use with established chemotherapy, which prompted our interest to select topoisomerase II as an interesting target for molecular docking to support the biological results, as ETO action is based on the inhibition of topoisomerase II.

One of the vital points of the present research work is to determine the weight and volume of solid tumors, which subcutaneously formed on the left thighs of female albino mice after subcutaneous injection of 2.5 million cells of Ehrlich ascites carcinoma (EAC) per mouse for 2 weeks and then the mono or dual treatment by APAN or/and ETO for another 2 weeks. The resulting data showed a significant decrease in tumor weight and volume after the treatment by APAN or/and ETO, which may be due to the ability of APAN to intercalate into GC-rich sequences and encroach with the catalytic TOP2 activity and then induce apoptosis and stop cell cycle in the malignant cells. Neocryptolepine as cancer chemotherapy is based on its capacity to inhibit TOP2 and produce enzyme-mediated DNA damage [41,42]. This agrees with [13], who used another analog of neocryptolpine at a dose of 100 mg/kg daily for five consecutive days and found that it affected the tumor volume. Further, the ETO improvement is due to ETO changes over TOP2 into a toxic substance that presents large amounts of transient protein-related breakdowns in the genome of treated cells [43]. While the best result was found when both APAN+ETO were used together, as APAN works to enhance the anti-tumor effect of ETO, which was noticeable in tumor weight and volume as compared to that of the EST group. These results suggested that APAN demonstrated promising effects to use with other anti-tumor medicine and could serve as the basis for further structure optimization to get effective and safe pharmacological compounds. These discoveries were inconsistent with Gupta [44], who found that the combination of etoposide (30 or 60 mg/kg b.w.) and 2-deoxy-D-glucose (2-DG, 2 g/kg b.w), which also work on TOP2 like APAN, should be useful in refining the side effect of ETO as it boosts the local tumor control, lacking any noteworthy adjustment in normal tissue toxicity.

Many histopathological abnormalities were observed in the skeletal muscle of the left thigh of the lower limb and the liver of mice bearing solid tumors, including cell degeneration, cellular inflammatory infiltration, and tumor cell aggregation. On the other hand, the mono treatment with APAN or ETO groups showed significantly decreased changes and observed other abnormalities, such as necrotic area, intracytoplasmic vacuolation, and aggregates of macrophages (giant cell). Moreover, the dual treatment with both APAN and ETO nearly restored the normal structure as compared to the EST and mono treatment groups. APAN or/and ETO blend therapy formed a substantial zone of necrosis in the tumor and aggregates of macrophages, indicating that APAN and ETO induced death of the tumor cells. This result agreed with that of El-Naa [45], who recorded that sildenafil, cisplatin, and their combination therapy produced a substantial area of necrosis in the tumor; these results indicated that sildenafil potentiates the antitumor activity of cisplatin via inducing death and proliferation inhibition of the Ehrlich tumor cells. These hepatic abnormalities during tumor growth might be due to the excessive production of reactive oxygen species (ROS) that lead to oxidative stress [46]. Free radicals induce severe changes in cell elements caused by fat peroxide, leading to cell membrane and nucleus damage, which results in the cell structure becoming abnormal and eventually achieving necrosis [46]. 

There were differences in the liver histology of the different groups under this study; a few of them experienced degeneration in the treated group, while hepatocytes of mice bearing tumors after treatment with APAN or/and ETO appeared with fewer cytoplasmic vacuoles than the EST group. This result agreed with that of Amin et al. [47] (2018), who mentioned that supplementation with l-arginine or omega-3 separately or together in rats treated with sodium valproate showed a noteworthy improvement in histopathological changes with nearly normal lobular architecture and hepatocytes with regular-sized nuclei, excluding the minor central veins and sinusoids that displayed congestion. APAN or/and ETO histopathology testing reduced the mean score of liver cell deterioration and inflammation between treatment groups, similar to that reported by Singh et al. [48] that l-arginine amended the hepatic cell resistances to be damaged by the lethal dosage of paracetamol. Grape seed extract modulated the effects of EST on kidney structure and functions [49].

The present results presented that APAN or/and ETO together thwarted oxidative stress indicators made via EST. These results establish the antitumor activity of APAN in the Ehrlich solid carcinoma in an in vivo prototype and this promising effect was complemented by APAN and ETO inhibition. In agreement with this, Neamatallah et al. [50] mentioned that nano ellagic acid improved the antitumor activity of cisplatin in the Ehrlich solid carcinoma and reduced its toxicity by the inhibition of the organic anion transporters 1 and 3, which are known to participate in the absorbance of cisplatin into renal tubular cells.

The current immunohistochemical results reported that the CD68, TNF-α, and survivin were strongly expressed in the solid tumor and liver tissue of EST animals and were markedly downregulated after EST was treated with mono and/or dual treatments in comparison with the EST mice. The maximum decrease was seen in the dual treatment group compared with EST and mono treatment groups. CD68 is connected to the family of lysosomal-associated membrane proteins and is famous for being a routine immune marker of cells of monocyte/macrophage lineage use, which has various functions in immunity, tumor biology, and inflammation [51]. In patients with hepatocellular carcinoma, the predictive power of the immunoexpression of Kupffer cells plays a vital role in different disease phases, and tumor formation has been associated with macrophage-mediated chronic inflammation [52]. Glycated Whey Protein (GWP) increased the pro-inflammatory cytokine expression as well as the phagocyte activity in RAW264.7 macrophages [53]. TNF-α is an exceptionally pleiotropic cytokine that has a crucial job in protection, insusceptible homeostasis, and irritations and plays an important role as an inflammation mediator associated with carcinogenesis and tumor progression [54]. Turculeanu et al. [55] reported a high level of TNF-α in tonsil carcinoma, signifying that this cytokine may be produced via other cells existing in the tumor microenvironment, counting the tumor cells themselves. The level of serum TNF-α was positively associated with a soaring frequency of post-treatment distant metastases and bone invasion in patients with nasopharyngeal carcinoma [56]. Additionally, these outcomes concurred with those of Aldubayan et al. [57], who detailed that EST incited a huge increment in TNF-α in serum and tumor tissues; this expansion in TNF-α level in the tumor-distressed mice could be because of an ascent in macrophage-delivered ROS, which builds lipid peroxidation. TNF-α and IL-1β were released from keratinocytes primarily affected by ribotoxic stress and involved in apoptosis [58]. The current results agree with that of Ma et al. [59], who reported that neocryptolepine derivative CFNC inhibits TNF-α and topoisomerase in gastric cancer. Suppression of apoptosis is important for carcinogenesis, and restriction of apoptosis protein survivin in a tumor gene is of critical importance for the regulation of cell division, survival, embryonic development, and tumor biology [60]. Tumor growth tissues showed positive expression for survivin, while non-tumor tissues displayed little noticeable staining by immunohistochemistry [40]. Melatonin downregulated the expression of survivin to prevent cell growth, survival, and metastasis of solid tumors [61].

The present study showed a noteworthy increase in serum AFP, CEA, and anti-dsDNA levels in the EST group in comparison with those of the control group. Treatments of EST with APAN or ETO exhibited a significant reduction in AFP, CEA, and anti-dsDNA in comparison with those of the EST group; in contrast, the treatment with both APAN and ETO almost normalized AFP, CEA, and anti-dsDNA, which proves that APAN has an anti-inflammatory effect and works well in co-treatment. Current results agree with that of El-Masry et al. [4], who detected a significant increase in the levels of anti-dsDNA in the EST-bearing mice and a significant reduction succeeding treatment of the tumor with vitamin B17 at a dose of 175 mg/kg body weight/day for 2 weeks. 

While the progress of tumors in the body can disturb functions of the vital organs, the results of the existing study exposed that induction of Ehrlich solid tumor in mice induced hepatic dysfunctions, which were represented by a noteworthy increase in the serum levels of serum ALT, AST, and ALP and also a noteworthy decrease in total protein and albumin concentrations. The result of the present study agrees with that of Abd ELdaim et al. [49], who found that EST in mice brought on a change of liver function biomarkers by an elevation of serum ALT, AST, and ALP and reduction in serum total protein and albumin levels that might be accredited to the relative hypoxia in periventricular regions of the liver; other studies by Dolai et al. [62] and Abou Zaid [63] report that EAC mice exhibited raised activities of liver enzymes due to hepatocellular damages. 

Hepatic toxicity induced through tumor growth may be due to the extreme production of ROS that leads to oxidative stress and damage [46,64,65]. The present data indicated that treatment with APAN improved liver biomarkers, and when ETO was used, improvement also occurred. On the other hand, treatment with both APAN+ETO together is better than either of them used alone, as APAN improved the side effect of ETO. A study by El Nabi et al. [66] agrees with the finding in this study that ETO induced several side effects on rat liver and it is better to use another natural product such as ginger as co-treatment to reduce its harmful effect. Star anise protected the kidney from ETO side effects in male rats [67]. Resveratrol consumption as a phytochemical agent with anticancer drugs confirmed their immune-modulatory efficiency against liver cancer in rats [68].

## 5. Conclusions

The present study has investigated the synergistic antitumor effect of the natural alkaloid neocryptolepine analog (APAN) and etoposide (ETO) on EST. The tumor was treated with APAN, ETO, or a combination of both for 2 weeks. Compared to the treatment of APAN or ETO alone, increased antitumor activity was observed with the combination of both. Molecular docking studies were performed on both TNF-α and topoisomerase II in consistency with the biological testing. Detailed molecular interaction patterns demonstrated hydrogen bonding, *pi*-stacking, and *pi* hydrophobic interactions in addition to APAN recording promising binding affinities in both target proteins. These results suggest that the combination of aminoneocryptolepine and etoposide may be a synergistic therapeutic option for Ehrlich solid tumor-induced hepatic injury in female mice. 

## Figures and Tables

**Table 1:** Docking results of APAN at the target protein 2AZ5.

Target	Protein Code	Binding Affinity	RMSD	Residues Involved in Interaction
TNF-α	2AZ5	−5.0704	2.08865	TYR 151: H-donor TYR 59: *π* stacking (*pi*-cation). TYR 119: *π–π*
Topoisomerase II	3FOE	−5.5996	1.1698	DG: H-donor DG: Ionic DA: H–*pi* *DG: π–π* *DG: π–π* *DG: π–π*

**Table 2:** Etoposide or/and APAN effect on tumor weight and volume at the end of different treatments.

Experimental Groups	Tumor Weight (g)	Tumor Volume (mm^3^)	Reduction Ratio (%)
EST	1.2475 ± 0.0894	1.49 ± 0.23	—
EST+APAN	0.6367 ± 0.0410 *	1.21 ± 0.36	19.8 *
EST+ETO	0.5775 ± 0.0406 *	1.06 ± 0.41	29.9 *
EST+APAN+ETO	0.2522 ± 0.0086 **	0.82 ± 0.40	45 **

Values are expressed as mean ± SE; n = 8 mice for each group; means in each column with a significantly different values denoted by (*); (**) indicates the maximum decrease ratio; *p* < 0.01.

**Table 3:** Histopathological and immunohistochemical changes in Ehrlich tumor and hepatic tissues.

Alterations	EST	EST+APAN	EST+ETO	EST+APAN+ETO
Cell degeneration	++++	++	+++	+
Necrosis	++++	++	+++	+
Cellular inflammatory infiltration	++	++	+++	+
Tumor cell	++++	+++	++	+
Intracytoplasmic vacuolation	++	+	+++	+
Giant cell	+++	++	+	+
Immuno-expression of CD68	++++	++	+++	+
Immuno-expression of TNF-α	++++	+++	++	+
Immuno-expression of survivin	++++	+++	++	+

Data were expressed as: (+) slight, (++) mild, (+++) moderate, and (++++) severe. The significance was detected via using one-way ANOVA, then followed by the Tukey-HSD test.

**Table 4:** Changes in serum tumor markers.

	Parameters	AFP (ng/mL)	CEA (ng/mL)	Anti-dsDNA (U/mL)
Groups				
Control		0.1043 ± 0.0156	0.3046 ± 0.0398	9.7937 ± 0.2028
APAN		0.2030 ± 0.0065	0.3454 ± 0.0171	16.9925 ± 1.5185
ETO		0.1341 ± 0.0113	0.1829 ± 0.0120	12.0663 ± 1.9776
APAN+ETO		0.2203 ± 0.0053	0.2526 ± 0.0159	16.7925 ± 1.3601
EST		0.9876 ± 0.0430 *	1.2844 ± 0.1128 *	36.2700 ± 1.7377 *
EST+APAN		0.3946 ± 0.0349 **	0.5754 ± 0.0319 **	17.1512 ± 1.8841 **
EST+ETO		0.3628 ± 0.0400 **	0.4975 ± 0.0195 **	16.1287 ± 1.1369 **
EST+APAN+ETO		0.1559 ± 0.0184 **	0.3011 ± 0.0429 **	13.7000 ± 0.5397 **

Data are presented as mean ± SE, n = 8. p < 0.01, (*) indicates a significant difference compared to the control group, and (**) indicates a significant difference compared to the EST group.

**Table 5:** Changes in serum liver function.

	Parameters	ALT (U/I)	AST (U/I)	ALB (gm/dl)	ALP (U/I)	Total Protein (gm/dL)
Groups						
Control		46.3750 ± 2.6693	142.7500 ± 3.2404	4.4175 ± 0.0604	128.5000 ± 4.6904	5.9962 ± 0.0427
APAN		66.3750 ± 2.6693	219.7500 ± 3.4949	3.2550 ± 0.0288	150.5000 ± 3.1623	6.3275 ± 0.0396
ETO		73.1000 ± 2.9799	240.3750 ± 3.3780	3.8125 ± 0.0396	161.8750 ± 3.3139	5.6188 ± 0.0494
APAN+ETO		75.2500 ± 3.1623	229.6250 ± 3.2486	3.3975 ± 0.0324	215.2500 ± 2.8158	6.0000 ± 0.0346
EST		94.2500 ± 4.4641 *	304.8750 ± 3.7961 *	3.4750 ± 0.0374 *	193.8750 ± 3.1820 *	5.0075 ± 0.0427 *
EST+APAN		90.8750 ± 4.0156 **	160.3750 ± 3.5026 **	3.6850 ± 0.0245 **	142.8750 ± 2.9001 **	5.5625 ± 0.0377 **
EST+ETO		78.3125 ± 3.1046 **	144.0000 ± 3.4641 **	3.5312 ± 0.0327 **	137.3750 ± 3.7009 **	5.3112 ± 0.03488 **
EST+APAN+ETO		53.8750 ± 4.4861 **	104.1250 ± 2.9001 **	3.7538 ± 0.0463 **	101.2500 ± 4.0620 **	5.5375 ± 0.0420 **

Data are present as mean ± SE, n = 8. *p* < 0.01, (*) indicates a significant difference in comparison to the control group, and (**) indicates a significant difference in comparison to the EST group. Aspartate aminotransferase (AST), alanine aminotransferase (ALT), alkaline phosphatase (ALP), albumin (ALB), and total protein.

## Data Availability

All data used in this study are included in this published article.
